# Potential higher risk of tethered spinal cord in children after prenatal surgery for myelomeningocele: A systematic review and meta-analysis

**DOI:** 10.1371/journal.pone.0287175

**Published:** 2023-06-28

**Authors:** Jochem K. H. Spoor, Charlotte C. Kik, Marie-Lise C. van Veelen, Clemens Dirven, Jena L. Miller, Mari L. Groves, Philip L. J. DeKoninck, Ahmet A. Baschat, Alex J. Eggink

**Affiliations:** 1 Department of Pediatric Neurosurgery, Erasmus MC Sophia Children’s Hospital, Rotterdam, The Netherlands; 2 Johns Hopkins Center for Fetal Therapy, Department of Gynaecology & Obstetrics, Johns Hopkins University, Baltimore, Maryland, United States of America; 3 Department of Neurosurgery, Johns Hopkins University, Baltimore, Maryland, United States of America; 4 Department of Obstetrics and Gynaecology, Division of Obstetrics and Fetal Medicine, Erasmus MC Sophia Children’s Hospital, Rotterdam, The Netherlands; Duke University Medical Center: Duke University Hospital, UNITED STATES

## Abstract

**Introduction:**

We performed a systematic review and meta-analysis on the incidence of secondary tethered spinal cord (TSC) between prenatal and postnatal closure in patients with MMC. The objectives was to understand the incidence of secondary TSC after prenatal surgery for MMC compared to postnatal surgery for MMC.

**Material and methods:**

On May 4, 2023, a systematic search was conducted in Medline, Embase, and the Cochrane Library to gather relevant data. Primary studies focusing on repair type, lesion level, and TSC were included, while non-English or non-Dutch reports, case reports, conference abstracts, editorials, letters, comments, and animal studies were excluded. Two reviewers assessed the included studies for bias risk, following PRISMA guidelines. TSC frequency in MMC closure types was determined, and the relationship between TSC occurrence and closure technique was analyzed using relative risk and Fisher’s exact test. Subgroup analysis revealed relative risk differences based on study designs and follow-up periods. A total of ten studies, involving 2,724 patients, were assessed. Among them, 2,293 patients underwent postnatal closure, while 431 received prenatal closure for the MMC defect. In the prenatal closure group, TSC occurred in 21.6% (n = 93), compared to 18.8% (n = 432) in the postnatal closure group. The relative risk (RR) of TSC in patients with prenatal MMC closure versus postnatal MMC closure was 1.145 (95%CI 0.939 to 1.398). Fisher’s exact test indicated a statistically non-significant association (p = 0.106) between TSC and closure technique. When considering only RCT and controlled cohort studies, the overall RR for TSC was 1.308 (95%CI 1.007 to 1.698) with a non-significant association (p = .053). For studies focusing on children up until early puberty (maximum 12 years follow-up), the RR for tethering was 1.104 (95%CI 0.876 to 1.391), with a non-significant association (p = 0.409).

**Conclusion and discussion:**

This review found no significant increase in relative risk of TSC between prenatal and postnatal closure in MMC patients, but a trend of increased TSC in the prenatal group. More long-term data on TSC after fetal closure is needed for better counseling and outcomes in MMC.

## Introduction

Tethered spinal cord (TSC) encompasses a range of clinical symptoms that arise from spinal cord traction, which can be attributed to underlying factors such as impaired energy metabolism, disrupted blood flow, and aberrant electrophysiological function resulting from cord stretching. Additionally, these symptoms may also arise as a consequence of scar tissue formation at the site of surgical intervention [1–4]. Primary tethering of the spinal cord is seen at birth in all patients with myelomeningocele (MMC) [5–7], while secondary re-tethering complicates 2.8–32% of surgical MMC closures [8–13]. The latter is the combination of the fixed position of the spinal cord and its predefined length thereby interfering with the physiological ascent in the spinal canal during the early years of development. Consequently, the first signs of retethering often occur during the period of rapid longitudinal growth, usually between 5 and 9 years of age [8, 13].

Tethered spinal cord can superimpose additional neurologic and structural disabilities on patients with MMC which can range from pain, lower limb weakness, gait disorders, neurogenic bowel and bladder to orthopedic abnormalities such as progressive scoliosis, leg-length discrepancy, foot asymmetry, and foot deformity [14–19]. Accordingly, tethered spinal cord has the potential for an independent significant impact on a wide range of outcomes that affect functionality and quality of life in patients with MMC.

Prenatal MMC closure is a more recently introduced treatment that has been shown to improve hindbrain herniation, decrease the need for postnatal shunting for hydrocephalus as well as improving motor function and neurodevelopmental outcomes. On the other hand, despite these beneficial effects, there appeared to be an increased need for secondary spinal cord untethering surgery before the age of 12-months and a higher incidence of dermoid cysts in infants that underwent prenatal surgery [20]. A recent literature review and evidence-based guideline concluded the presence of class II and III evidence demonstrating an equal or higher incidence of TSC developing after prenatal MMC closure [21]. However, no meta-analysis has been performed to weigh the outcome of retethering between prenatal and postnatal closure groups. Given the importance of spinal cord tethering in determining outcome of patients with MMC we aimed to perform a systematic review and meta-analysis to evaluate the current literature on the incidence of retethering between prenatal and postnatal closure in patients with MMC.

## Materials and methods

### Search strategy

This study was conducted in line with the PRISMA guidelines and registered in PROSPERO, (identification number 383940) [22]. We systematically searched the literature for primary intervention studies reporting on the incidence of symptomatic secondary TSC in the MMC population. The systematic search was performed using the search terms “spina bifida”, "spinal dysraphism", "tethered spinal cord", and synonyms in Pubmed, Medline, Embase, and the Cochrane Library on the 4^th^ of May, 2023.

### Study selection and eligibility criteria

Two investigators (C.C.Kik. and J.K.H.Spoor.) independently assessed the titles and abstracts to identify randomized clinical trials (RCTs) or cohort studies on the frequency of secondary TSC between prenatal and postnatal myelomeningocele repair. Primary studies reporting on type of repair, lesion level and TSC were included. Non-English or non-Dutch reports, case reports, conference abstracts, editorials, letters and comments, and animal studies were excluded. Subsequently, full texts were independently evaluated for eligibility following inclusion and exclusion criteria. The two investigators reviewed the titles and abstracts for relevance and identified citations for full-text review using the online reviewing tool Rayyan [23] (http://rayyan.qcri.org).

The occurrence of TSC was either defined by symptomatic TSC, which included neurologic or urological deterioration (i.e., new-onset upper urinary tract dilatation, decreased bladder compliance, incontinence, and gait deterioration) or by the necessity of surgical intervention for tethered cord release.

### Data extraction

Two investigators extracted data with any disagreement resolved by consensus. For each relevant study, the following data were collected: first author, year of publication, country of conduct, study design, number of patients in the intervention group (prenatal repair), number of patients in the control group (postnatal repair), the mean age of TSC diagnosis, gender, anatomical level of the defect, the incidence of symptomatic tethered cord syndrome, incidence of surgical intervention of symptomatic tethered cord syndrome.

### Quality assessment and publication bias

The Methodological Index for Non-Randomized Studies (MINORS) criteria is a validated tool used to critically appraise the methodologies of included studies [24]. MINORS assesses the presence of various forms of bias, including selection, performance, detection, attrition, reporting, and other types of bias, which are scored as ’not reported,’ ’reported but inadequate,’ or ’reported adequately.’ Two independent authors (JS and CK) scored all articles, with a third reviewer (PK) consulted in cases of disagreement, and the majority vote was used. The comparative studies were eligible for a maximum score of 24 points, whereas retrospective comparative studies were eligible for up to 16 points due to the inapplicability of the MINORS criteria for "an adequate control group" "contemporary groups" and "baseline equivalence of groups" and "adequate statistical analysis" in non-comparative designs. Publication bias was visually assessed using Deek’s funnel plots.

### Statistical analysis

The frequency of TSC among both MMC closure types was calculated, as well as the frequency of TSC in the prenatal and postnatal groups. The patients’ baseline characteristics were presented either by frequency or by sample mean and standard deviation. In cases where only the sample median was given, the estimated mean was calculated via the quantile estimation method [25]. The association between the occurrence of TSC and surgical closure technique was evaluated by calculating the relative risk and a Fisher’s exact test. Further subgroup analysis was performed to identify the difference in relative risk between study designs and follow-up periods. All analyses were performed using SPSS 28.0 (IBM Corp. Released 2021. IBM SPSS Statistics for Windows, Version 28.0. Armonk, NY: IBM Corp).

## Results

### Study identification and selection

**Fig 1:**
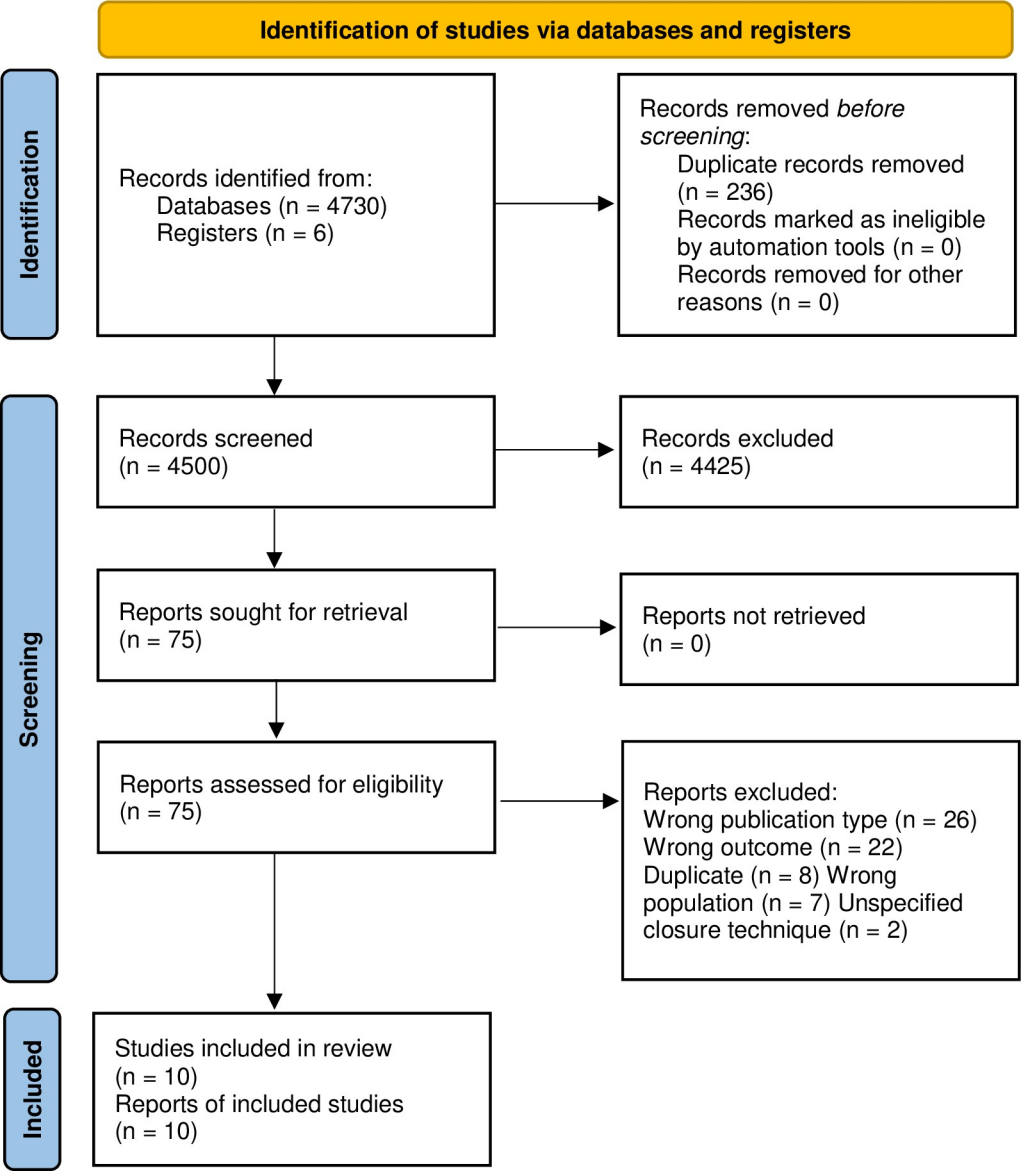
PRISMA flow diagram for study identification and selection.

The systematic search yielded 4736 articles. After removal of duplicates, 4500 articles were screened by title and abstract for eligibility. 75 articles were retrieved for full-text assessment and evaluated for inclusion. Sixty-five articles were excluded due to various reasons as outlined in Fig 1. Twenty-six studies were excluded on the basis of their publication type (i.e. abstracts or case-report studies), 22 for reporting a different outcome than TSC, 8 were duplicates, 7 concerned a population different from MMC patients and 2 did not specify which closure technique was used. Finally, 10 studies were included for quality assessment [9–13, 26–30].

### Study characteristics

The included studies were published between 2006 and 2021 with a mean study period of 13.9 ± 7.7 years (Table 1). The largest share of included studies were single center retrospective cohort studies (80.0%) [9–13, 26, 27, 30]. One randomized controlled trial and a prospective cohort with a historical control were also included [28, 29].

**Table 1:** Characteristics of the included studies.

					Study Period			
Author	Publication Date	Location	Study type	Temporality	From	Until	Closure type	TSC definition
Worley et al.	2021	Multicenter, USA	Cohort Study	Prospective with historic control	1997	2017	Prenatal and Postnatal	Surgical TSC release
Borgstedt et al.	2020	Aarhus, Denmark	Cohort Study	Retrospective	1996	2015	Postnatal	Surgical TSC release
Houtrow	2020	Multicenter, USA	Randomized Controlled Trial (Follow-Up)	Prospective	2011	2017	Prenatal and Postnatal	Surgical TSC release
Spoor et al.	2019	Rotterdam, The Netherlands	Cohort Study	Retrospective	2000	2018	Postnatal	Surgical TSC release
Kellogg et al.	2018	Pittsburg, USA	Cohort Study	Retrospective	1995	2015	Postnatal	Surgical TSC release
Beuriat et al.	2018	Lyon, France	Cohort Study	Prospective	2015	2016	Postnatal	Surgical TSC release
Bowman et al.	2009	Chicago, USA	Cohort Study	Retrospective	1975	2008	Postnatal	Symptomatic TSC
Danzer et al.	2008	Philadelphia, USA	Cohort Study	Retrospective	1998	2003	Prenatal	Surgical TSC release
Talamonti et al.	2007	Milan, Italy	Cohort Study	Retrospective	1980	2005	Postnatal	Symptomatic TSC
Tarcan et al.	2006	Istanbul, Turkey	Cohort Study	Retrospective	1996	2005	Postnatal	Symptomatic TSC

Seven studies utilized surgical TSC release as their primary outcome measure, whereas three studies focused on evaluating signs or symptoms associated with spina bifida (Table 1). For instance, Bowman et al. (2009) examined progressive scoliosis, decline in lower extremity motor strength, lower extremity contractures, spasticity in the lower extremities, gait changes, alterations in urological function, and/or back pain [13]. Talamonti et al. (2007) included patients exhibiting increased weakness, development of new neurological deficits, hypertonia and/or clonic movements, progressively worsening scoliosis and/or orthopedic anomalies, severe pain at the site of the back wound, and deterioration of urinary function [26]. Lastly, Tarcan et al. (2006) diagnosed TSC based on urological deterioration and neuro-orthopedic findings, without further specification [27].

In total, the included studies reported on the outcomes of 2724 patients, of whom 2293 underwent postnatal closure and 431 prenatal closure of the MMC defect. Of the included patients, 49% was male (Table 2). Most patients had a MMC at the lumbar level (n = 1039) followed by sacral (n = 372), thoracic (n = 138) and cervical lesions (n = 2), in 1173 patients the level was not specified. The prenatal closure group had 52.4% (n = 226) patients with lumbar MMC lesions, as compared to 35.4% of the postnatal closure group (n = 813) (Table 3). On average, patients presented with or were surgically treated for TSC at age 7.1 ± 1.7. The mean follow-up periods averaged between 4.0 and 11.8 years of age. Four postnatal studies (44.4%) with a total number of 507 patients did not include patients with TSC under the age of 2.5 years, while the majority of prenatal closure studies with a total number of 125 patients reported on patients with TSC before the first year of life [9, 10, 12, 26, 29, 30].

**Table 2:** Patient characteristics.

				Gender		Follow-up period (in years)				No. Tethering		Age of TSC presentation (years)			
Author	Publication Date	No Patients postnatal	No. Patients prenatal	Male	Female	Mean	Median	Minimum	Maximum	Postnatal	Prenatal	Mean	Median	Minimum	Maximum
Worley et al.	2021	648	298	468	478	3.95	NA	1.42	11.5	102	54	NA	NA	NA	NA
Borgstedt et al.	2020	166	0	82	84	NA	25	12	46	45	0	NA	12	5	15
Houtrow et al.	2020	82	79	74	87	NA	NA	6	10	12	23	NA	NA	NA	NA
Spoor et al.	2019	93	0	46	47	11.8	NA	1	18.2	11	0	8	NA	3	12
Kellogg et al.	2018	153	0	64	69	9.9	NA	2	20	24	0	NA	NA	NA	NA
Beuriat et al.	2018	46	0	22	24	8.1	NA	1	18	7	0	6.7	NA	2.8	13.6
Bowman et al.	2009	502	0	56	58	12	NA	1.1	11	114	0	7	NA	0.84	21.8
Danzer et al.	2008	0	54	NA	NA	NA	NA	NA	NA	0	16	3.1	2.3	0.3	7.8
Talamonti et al.	2007	202	0	101	101	9.3	NA	1	25	61	0	7.5	NA	4	25
Tarcan et al.	2006	401	0	NA	NA	NA	NA	NA	NA	56	0	5.8	4.1	0.3	15

NA = Not Available

**Table 3:** Location of the MMC lesions in the total, postnatal closure and prenatal closure patient population.

		MMC level total				MMC level postnatal				MMC level prenatal			
Author	Publication Date	Cervical	Thoracic	Lumbar	Sacral	Cervical	Thoracic	Lumbar	Sacral	Cervical	Thoracic	Lumbar	Sacral
Worley et al.	2021	0	91	590	265	0	71	414	163	0	20	176	102
Borgstedt et al.	2020	2	16	134	14	2	16	134	14	NA	NA	NA	NA
Houtrow et al.	2020	0	3	105	53	0	1	55	26	0	2	50	27
Spoor et al.	2019	0	19	63	11	0	19	63	11	NA	NA	NA	NA
Kellogg et al.	2018	0	9	126	18	0	9	126	18	NA	NA	NA	NA
Beuriat et al.	2018	NA	NA	21	11	NA	NA	21	11	NA	NA	NA	NA
Bowman et al.	2009	NA	NA	NA	NA	NA	NA	NA	NA	NA	NA	NA	NA
Danzer et al.	2008	NA	NA	NA	NA	NA	NA	NA	NA	NA	NA	NA	NA
Talamonti et al.	2007	NA	NA	NA	NA	NA	NA	NA	NA	NA	NA	NA	NA
Tarcan et al.	2006	NA	NA	NA	NA	NA	NA	NA	NA	NA	NA	NA	NA

NA = Not Available

### Quality assessment

On average, two studies scored relatively high on the MINORS criteria, with scores of 18 and 21 out of 24 respectively, due to their prospective and comparative design. For the six studies that had a non-comparative design, the average MINORS score was 7.5 out of 16, with a standard deviation of 2.3 (Table 4). In general, the majority of studies were deemed inadequate or had limited assessment in terms of prospective determination of sample size and loss to follow-up, primarily attributable to the utilization of a retrospective study design. Studies that scored lower on the “unbiased assessment of the study endpoint” were those that included symptomatic TSC as primary endpoint instead of surgical TSC release.

**Table 4:** MINORS quality assessment for all included studies.

**Criteria**	Worley et al. 2021	Borgstedt et al. 2020	Houtrow et al. 2020	Spoor et al. 2019	Kellogg et al. 2018	Beuriat et al. 2018	Bowman et al. 2009	Danzer et al. 2008	Talamonti et al. 2007	Tufan Tarca et al. 2006
A clearly stated aim	2	2	2	2	2	2	2	2	2	2
Inclusion of consecutive patients	0	0	2	0	0	2	0	0	0	0
Prospective collection of data	2	0	2	0	0	2	0	0	0	0
Endpoints appropriate to the aim of the study	2	2	2	2	2	2	2	2	2	2
Unbiased assessment of the study endpoint	2	2	2	2	2	2	1	1	1	1
Follow-up period appropriate to the aim of the study	2	2	2	2	2	2	1	1	1	1
Loss to follow-up less than 5%	0	0	1	0	0	0	0	0	0	0
Prospective calculation of the study size	0	0	0	0	0	0	0	0	0	0
**Additional criteria for comparative studies**										
An adequate control group	2	0	2	0	0	0	0	0	0	0
Contemporary groups	2	0	2	0	0	0	0	0	0	0
Baseline equivalence of groups	2	0	2	0	0	0	0	0	0	0
Adequate statistical analyses	2	0	2	0	0	0	0	0	0	0
**Total MINORS score**	**18**	**8**	**21**	**8**	**8**	**12**	**6**	**6**	**6**	**6**

All items are scored 0 (not reported/not applicable), 1 (reported but inadequate) or 2 (reported and adequate). Scores range from 0-of the MINORS-tool range from 0–24 for comparative studies and 0–16 for non-comparative studies.

### Tethered spinal cord

In the prenatal closure group, the frequency of TSC was 21.6% (n = 93), as compared to 18.8% (n = 432) in the postnatal closure group. The relative risk (RR) of TSC in patients with prenatal MMC closure compared to postnatal MMC closure was 1.145 (95%CI 0.939 to 1.398). There was a statistically non-significant association between TSC and closure technique as assessed by Fisher’s exact test, *p* = 0.106. A further subgroup analysis was done to evaluate the difference between study types, duration of follow-up, and study period. When comparing study types, the overall RR for TSC when evaluating only the RCT & controlled cohort studies was 1.308 (95%CI 1.007 to 1.698) with a statistically non-significant association, *p* = .053 [28, 29]. In studies restricted to children up to the early stages of puberty, with a maximum follow-up duration of 12 years, the analysis revealed a relative risk (RR) of 1.104 (95% CI 0.876 to 1.391) for TSC, with a statistically non-significant association, *p* = 0.409 [13, 28, 29].

## Discussion

This study indicates a potential modest elevation in the relative risk of tethered spinal cord (TSC) among individuals who undergo prenatal closure for myelomeningocele (MMC), in comparison to those who undergo postnatal closure; however, this association does not reach statistical significance. Upon careful evaluation of the largest comparison groups derived from randomized controlled trials and prospective cohort studies, a trend of increased relative risk of TSC becomes apparent in the prenatal closure group, albeit without attaining statistical significance.

While it may be assumed that fetuses heal with less scarring, and therefore prenatal closure may seem like a preferable option, it is worth noting that skin healing in fetuses after 24 weeks of gestation is thought to be histologically identical to that in adults [31–34]. This is significant because fetal surgery for MMC typically takes place around the 24th week of gestation. As a result, it is possible that skin healing is not different between prenatal and postnatal closure, and therefore the effect on retethering may also be similar.

It is important to consider that the surgical techniques used in prenatal closure may differ from those used in postnatal closure, such as related to the re-tubulation of the placode, which is commonly done in postnatal surgery but not always possible in prenatal surgery [20]. This may help explain the slightly higher relative risk of rethetering in fetal closure for MMC. In addition, the neurosurgical part of prenatal surgery is continuously evolving, largely driven by the development of a fetoscopic approach [35, 36]. Nevertheless, it is important to acknowledge the higher incidence of TSC, and we emphasize the necessity of further studies specifically focusing on this aspect of postnatal outcomes as it provides important information for preoperative counselling. However, it is also essential to guide future efforts in optimizing the prenatal closure technique.

There exist several limitations to this study that warrant consideration. First, the postnatal closure patient cohort was found to be 5.3 times larger than the prenatal closure cohort. The number of subjects in the prenatal cohorts was relatively small, and only a single randomized controlled trial (RCT) has been conducted thus far. Additionally, the majority of centers did not match the surgical site for the outcome. A recent investigation conducted by Dias et al. (2021) examining TSC incidence demonstrated considerable variability in TSC occurrence across the participating centers [37]. They utilized data obtained from the National Spina Bifida Registry, which is housed at the Center for Disease Control and Prevention in the United States. When assessing the overall data, it is important to consider that while all the multicenter studies included in this meta-analysis were matched for surgical site characteristics, this factor still poses a concern. The present study encounters a notable limitation stemming from the unfeasibility of categorizing or partitioning the available data. This limitation arises due to several factors, including the coexistence of potential indications for TSC release or the difference in aggressiveness of TSC approach, such as the routine combination of TSC treatment with scoliosis surgery. Moreover, it is conceivable that improved cognitive outcomes resulting from prenatal interventions may contribute to an enhanced detection rate of TSC cases.

Furthermore, there is a significant selection bias to be considered. Not all patients qualify for prenatal closure and might be excluded due to the severity or location of the defect or maternal morbidities. The limited availability of RCTs means that the majority of included studies are composed of retrospective cohorts. Although it is known that surgical cohort studies such as “natural experiments” are not necessarily of a lesser methodological quality than surgical RCTs, there is a risk of performance bias [38–40]. Due to the strict maternal selection criteria for prenatal surgery, there is possible risk of selection bias towards mothers with a lower socioeconomic status in cohort studies as compared to RCTs. Finally, follow-up periods for prenatal groups vs. postnatal groups differ dramatically. Where retrospective studies on postnatal closure have follow-up periods of over 20 years, prenatal closure generally only reports on the first 3 to 4 years of a patient’s life, with one study reporting on a follow-up of up to 10 years [29]. These aforementioned factors collectively underscore the substantial constraint imposed on the current study.

This shorter follow-up raises an interesting question regarding the incidence and risk of developing TSC. Previous studies on TSC following postnatal MMC closure have shown that most patients undergo tethered cord release between the age of 9 and 15 [9]. The pathophysiology of increased strain on the spinal cord during a period of rapid growth results in an increase of symptomatic TSC [1, 2, 21, 41]. In postnatal closure studies, tethering before the first year of life is very uncommon. The first successful prenatal MMC closure was performed in 1997, and thus very few prenatal patients in that study have reached adulthood at this point in time [42]. Therefore, long-term results or complications remain unclear. It has been proposed that TSC occurs at a younger age in the prenatal closure group, while the absolute number of TSC remains equal to that of postnatal closure groups [30]. Interestingly, the study by Dias et al. (2021) on the occurrence of TSC in all spina bifida patients did not find an elevated frequency at puberty, but a relatively equal distribution per age [37]. With puberty in foresight, it is thus not particularly clear whether the prenatal group might be at risk of reoccurring tethering with the need for multiple surgical interventions, of which each carries a significant risk of further neurological deterioration.

In the end, there is a need for more follow-up data on the patients who have undergone prenatal MMC closure since the early beginning of this century. During prenatal MMC counselling, the direct functional outcomes and the possible need for future surgical interventions are important factors to consider when discussing the impact of MMC.

## Conclusions

This meta-analysis and systematic review shows that the relative risk of TSC is not significantly increased in the prenatal closure group compared to the postnatal group in MMC patients, although there is a trend of increased TSC in het prenatal closure group. In order to improve counselling on and the outcome of MMC, more long-term data on TSC after fetal closure for MMC is needed.

## Supporting information

S1 Checklist PRISMA 2020 checklist. (DOCX)

S2 Checklist PRISMA 2020 for abstracts checklist. (DOCX)

## Abbreviations

MMC: myelomeningocele
RR: relative risk
CI: confidence interval
TSC: tethered spinal cord
MINORS: Methodological Index for Non-Randomized Studies
